# Resolve and dissolve—An ultrasound‐guided investigation on the effects of hyaluronidase on different soft tissue fillers

**DOI:** 10.1111/jocd.16393

**Published:** 2024-05-20

**Authors:** Bruna S. F. Bravo, Thamires Cavalcante, Camila Silveira, Leonardo G. Bravo, Maria Carolina Zafra, Mariana Calomeni

**Affiliations:** ^1^ Department of Dermatology Bravo Private Clinic Rio de Janeiro Brazil

**Keywords:** dissolving, hyaluronic acid, hyaluronidase, injection, soft tissue fillers

## Abstract

**Background:**

Nonsurgical cosmetic procedures, particularly the use of hyaluronic acid (HA)‐based soft tissue fillers, are becoming increasingly popular. This trend has catalyzed the development of a plethora of HA‐based products differing in product characteristics, thereby catering to an ever‐widening spectrum of aesthetic applications. However, complications rise concomitant with the increasing number of procedures. Among the strategies to manage such adverse events is the enzymatic breakdown with hyaluronidase.

**Objective:**

To analyze the response of different HA‐based soft tissue filler materials to hyaluronidase injections.

**Methods:**

A total of 11 different HA‐based soft tissue fillers were evaluated using noninvasive ultrasound imaging to assess their behavior in response to hyaluronidase injections. The HA‐based soft tissue fillers were categorized according to their product characteristics into a structuring, volumizing, and lip volumizing group. Standardized injections of 0.2 cc were performed in chicken breast to simulate human tissue. Ultrasound measurements of width, height, and calculated volume were performed immediately after filler injection, 1 h and 24 h following hyaluronidase injection.

**Results:**

Regardless of the soft tissue filler analyzed, the most significant volume reduction occurred within the first h after applying hyaluronidase, with a 64.1% decrease from the initial volume. After 24 h, the total volume reduction reached 81.7%. No statistically significant differences were found when comparing the three groups at each follow‐up time period, except for the height measurement after 1 h. While width was statistically significant in all groups between the investigated follow‐up groups, the volume reduction was only statistically significant in the groups with the highest and second highest G' values (i.e., Group 1—structuring, Group 2—volumizing).

**Conclusion:**

The effectiveness of hyaluronidase in dissolving HA‐based fillers is initially independent of product characteristics of HA‐based fillers such as G‐prime, with increased efficacy in fillers with higher G‐prime values, as evidenced by significant volume reductions in such groups.

## 1 INTRODUCTION

The Global Survey from the International Society of Aesthetic Plastic Surgery (ISAPS) reveals a growing popularity of aesthetic procedures. In 2021 alone, over 5 000 000 treatments of hyaluronic acid (HA)‐based soft tissue fillers were performed globally, marking a significant increase of more than 60% compared to 5 years earlier in 2017. This surge starkly contrasts with the modest 7.3% rise in surgical procedures in the head and neck area during the same period, thereby underscoring a growing preference for nonsurgical alternatives.^1^ Patients are increasingly drawn to nonsurgical treatments such as HA‐based fillers for a variety of reasons including their minimal invasiveness, affordability, and quick recovery times.^2^, ^3^, ^4^, ^5^

In response to this ever‐growing demand, the aesthetic industry is heavily investing in the development and improvement of various HA‐based fillers. These products are tailored to specific purposes, differing in characteristics such as G prime (elastic modulus or gel hardness), HA particle size, HA concentration, gel cohesivity, degree of HA crosslinking, crosslinking conditions such as temperature and pH, and postcrosslinking modifications.^6^, ^7^ These characteristics have different clinical implications: for example, HA‐based fillers with greater ratios of crosslinked to noncrosslinked HA have been reported to be more resistant to enzymatic degradation and exhibit superior rheologic properties.^8^, ^9^, ^10^, ^11^ In vitro measurements of rheologic and physicochemical characteristics of soft tissue fillers allow for their comparison. The clinically most important ones are the complex modulus (G*; measuring the overall viscoelastic properties), the elastic modulus (G'; measuring the elastic properties), the viscous modulus (G"; measuring the viscous properties), and tan delta (δ; ratio between the viscous and elastic properties). Most commonly, G' is used as the main product characteristic of soft tissue fillers as it measures the ability to withstand deformation and regain its original shape after deformation, directly translating into clinical practice.^6^, ^12^

Together with the applied injection technique, these product characteristics determine how the soft tissue fillers inter‐ and react with the tissue and may significantly affect clinical outcomes.^8^ For instance, high G' soft tissue fillers are stiffer and can tolerate compression by the surrounding tissue, rendering them more prone to forming visible and palpable nodules if injected superficially.^6^ In such cases, another key advantage of these nonsurgical HA‐based options comes into play: their reversibility. HA, a glycosaminoglycan capable of retaining water in tissue, can be enzymatically dissolved via hyaluronidase. This enzyme, which can act as either hydrolase or lyase, targets specific bonds in the HA structure, thereby dissolving the substance and restoring the preinjection look.^13^, ^14^, ^15^

Despite extensive research on the behavior of HA‐based fillers in tissue, there is a paucity of studies elucidating the interaction between hyaluronidase and HA‐based fillers. This study aims to address this knowledge gap by investigating the response of different HA‐based soft tissue filler materials to hyaluronidase injections, thereby ultimately aiming to optimize safety and effectiveness of HA‐based soft tissue filler treatments, particularly in facial injections.

## 2 MATERIALS AND METHODS

### 2.1 Study setup

This interventional study investigated the behavior of different HA‐based soft tissue fillers in response to hyaluronidase using noninvasive ultrasound imaging. The study was conducted between September and October 2023 in the private practice of the senior author, Bravo Clinic in Rio de Janeiro, Brazil. A filler amount of 0.2 mL was injected into chicken breast pieces to simulate human tissue. All chicken breasts were then stored on a tray on the counter in an air‐conditioned room in the study center at a temperature of 22°C. In this study, a total of 11 commercially available HA‐based soft tissue fillers were investigated. Depending on their primary indication, these fillers were categorized into the following three groups:

- *Group 1*—“*Structuring*”: Juvedérm Volux (Allergan Aesthetics An AbbVie company, Dublin, Ireland); Belotero Intense (Merz Aesthetics, Frankfurt am Main, Germany); Restylane Lyft (Galderma, Fort Worth, TX, USA); and Rennova Lift Plus (Rennova, Goiânia, Brazil) (Figure 1)
- *Group 2*—“*Volumizing*”: Juvedérm Voluma (Allergan Aesthetics An AbbVie company, Dublin, Ireland); Belotero Volume (Merz Aesthetics, Frankfurt am Main, Germany), Restylane Volume (Galderma, Fort Worth, TX, USA); and Rennova Ultra Volume (Rennova, Goiânia, Brazil) (Figure 2)
- *Group 3*—“*Lip Volumizing*”: Juvedérm Volift (Allergan Aesthetics An AbbVie company, Dublin, Ireland); Belotero Intense (Merz Aesthetics, Frankfurt am Main, Germany); Restylane Kysse (Galderma, Fort Worth, TX, USA); and Rennova lips (Rennova, Goiânia, Brazil) (Figure 3)

**FIGURE 1:**
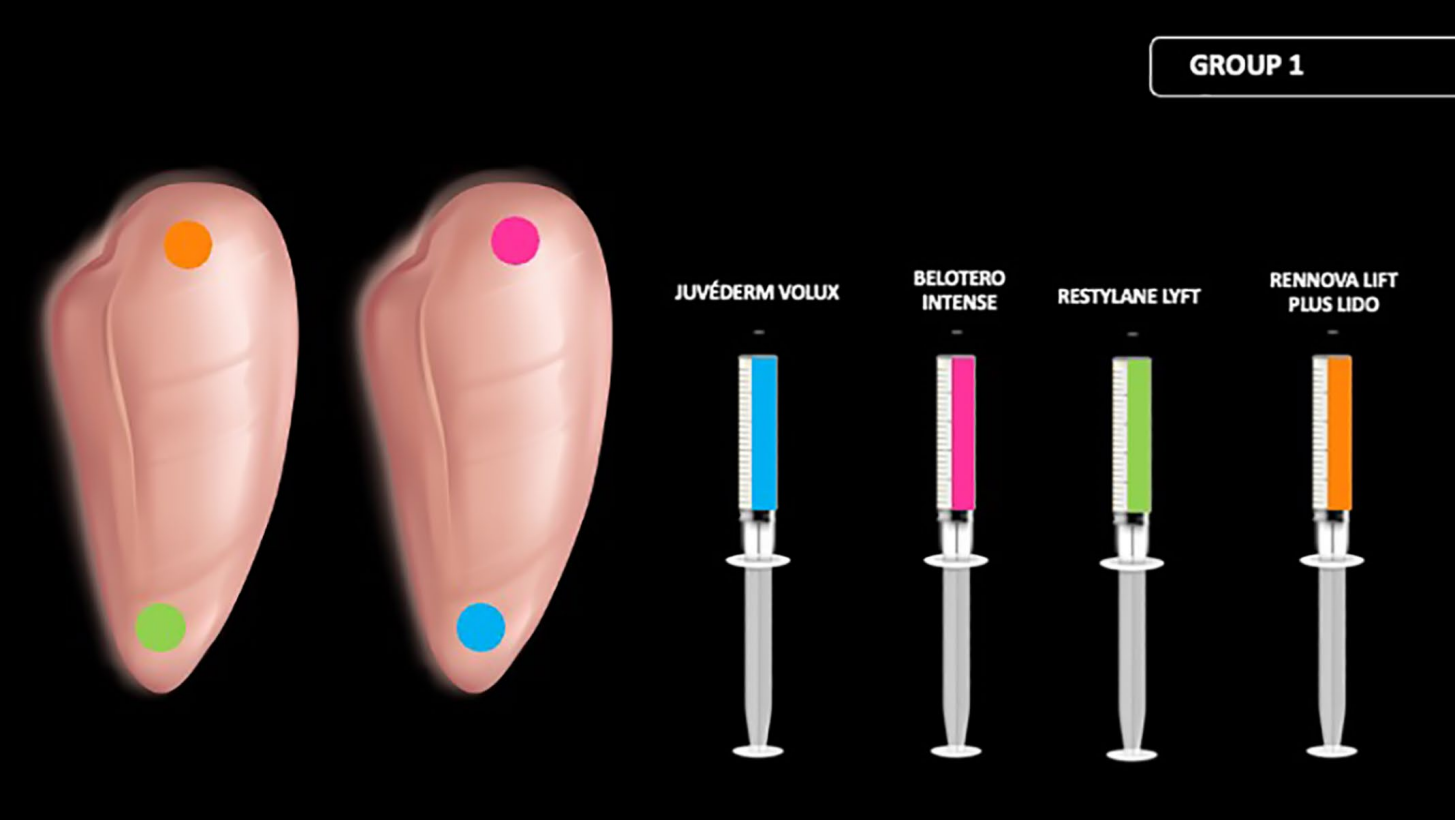
Illustration showing the study's methodology and placement of four different soft tissue fillers in the chicken breast. Group 1–Structuring comprised the following products: Juvedérm Volux (Allergan Aesthetics An AbbVie company, Dublin, Ireland); Belotero Intense (Merz Aesthetics, Frankfurt am Main, Germany); Restylane Lyft (Galderma, Fort Worth, TX, USA); and Rennova Lift Plus (Rennova, Goiânia, Brazil).

**FIGURE 2:**
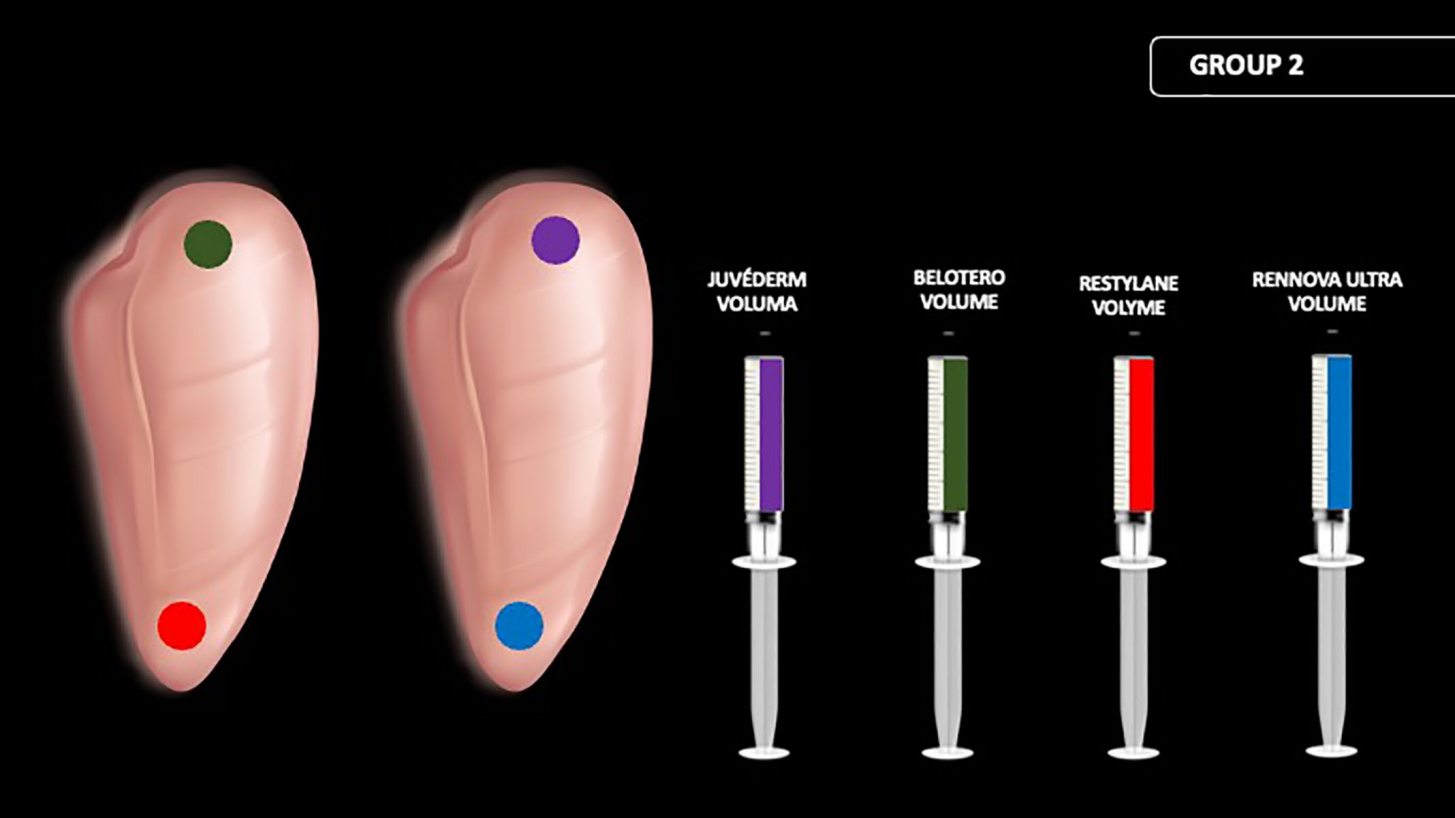
Illustration showing the study's methodology and placement of four different soft tissue fillers in the chicken breast. Group 2–Volumizing comprised the following products: Juvedérm Voluma (Allergan Aesthetics An AbbVie company, Dublin, Ireland); Belotero Volume (Merz Aesthetics, Frankfurt am Main, Germany), Restylane Volume (Galderma, Fort Worth, TX, USA); and Rennova Ultra Volume (Rennova, Goiânia, Brazil).

**FIGURE 3:**
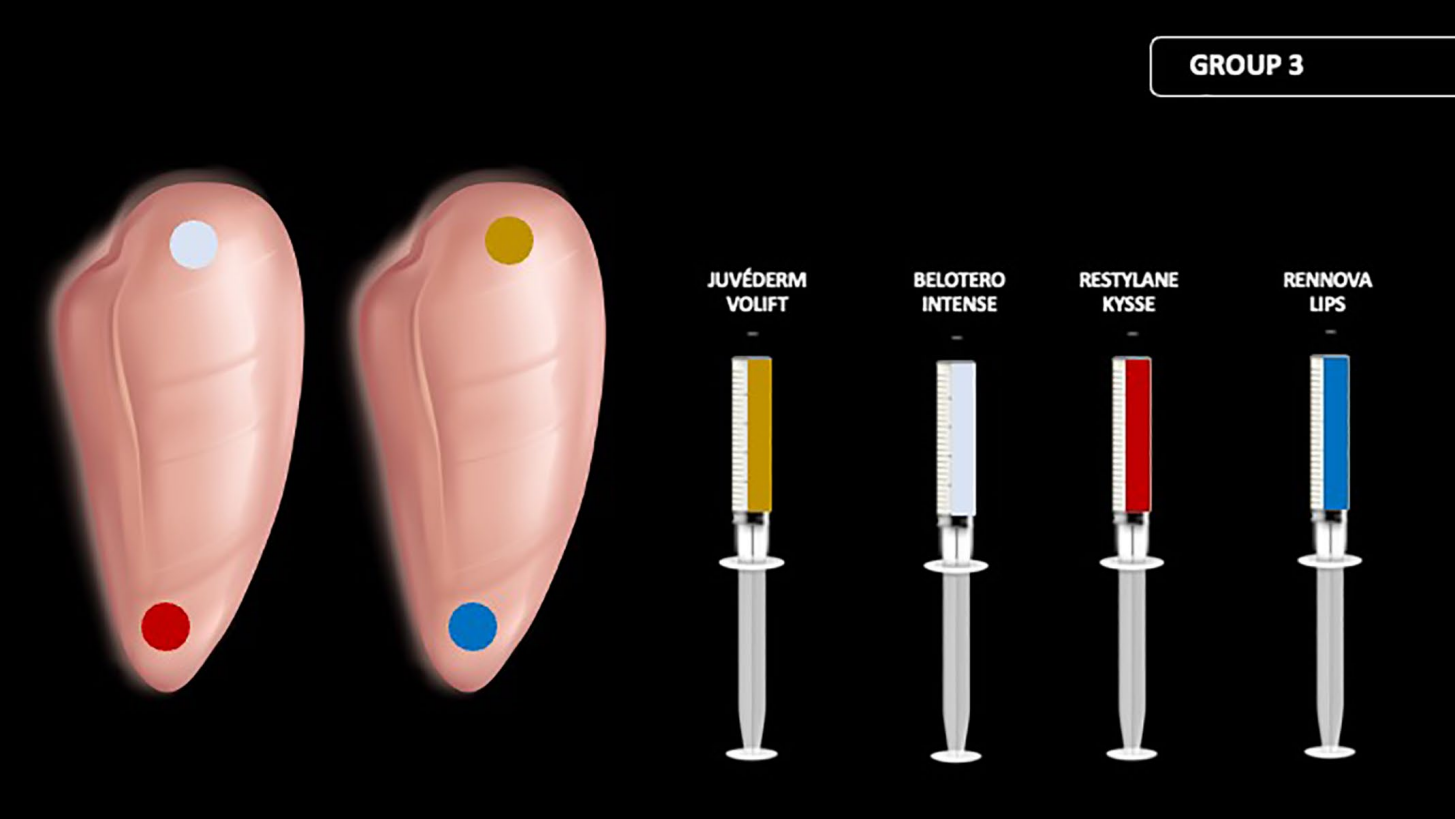
Illustration showing the study's methodology and placement of four different soft tissue fillers in the chicken breast. Group 2–Lip Volumization comprised the following products: Juvedérm Volift (Allergan Aesthetics An AbbVie company, Dublin, Ireland); Belotero Intense (Merz Aesthetics, Frankfurt am Main, Germany); Restylane Kysse (Galderma, Fort Worth, TX, USA); and Rennova lips (Rennova, Goiânia, Brazil).

The G' values for each soft tissue filler are summarized in Table 1.

**TABLE 1:** Summary of the G' values of each soft tissue fillers investigated in this study.

Group 1—“structuring”				Group 2—“volumizing”				Group 3—“lip volumizing”			
Juvedérm volux	Belotero intense	Restylane lyft	Rennova lift plus	Juvedérm voluma	Belotero volume	Restylane volume	Rennova ultra volume	Juvedérm volift	Belotero intense	Restylane kysse	Rennova lips
665 Pa^6^	255 Pa^6^	977 Pa^6^	414 Pa**	398 Pa^6^	438 Pa^6^	239 Pa^6^	251 Pa**	340 Pa^6^	255 Pa^6^	236 Pa^6^	*
⌀ 578 Pa				⌀ 332 Pa				⌀ 277 Pa			

*Note*: *No data on G prime values available; ** Information provided by the company.

Immediately after the injection, a baseline ultrasound examination was performed for each product. Thereafter, a consistent amount of 200 units of hyaluronidase (Toskani, Santa Eulalia de Ronsana, Barcelona, Spain) diluted in 0.2 mL of saline solution was injected into each HA bolus under ultrasound guidance. Follow‐up ultrasound investigations were conducted after 1h and 24 h by the same investigator.

### 2.2 Ultrasound imaging

At each ultrasound examination (i.e., immediately after the soft tissue filler injection, 1 h after the injection of hyaluronidase, and 24 h after the injection of hyaluronidase), the following measurements were taken three times for each HA bolus for accuracy:

- Width (in mm): maximum horizontal extent of the injected HA bolus
- Height (in mm): maximum vertical extent of the injected HA bolus (Figure 4)

**FIGURE 4:**
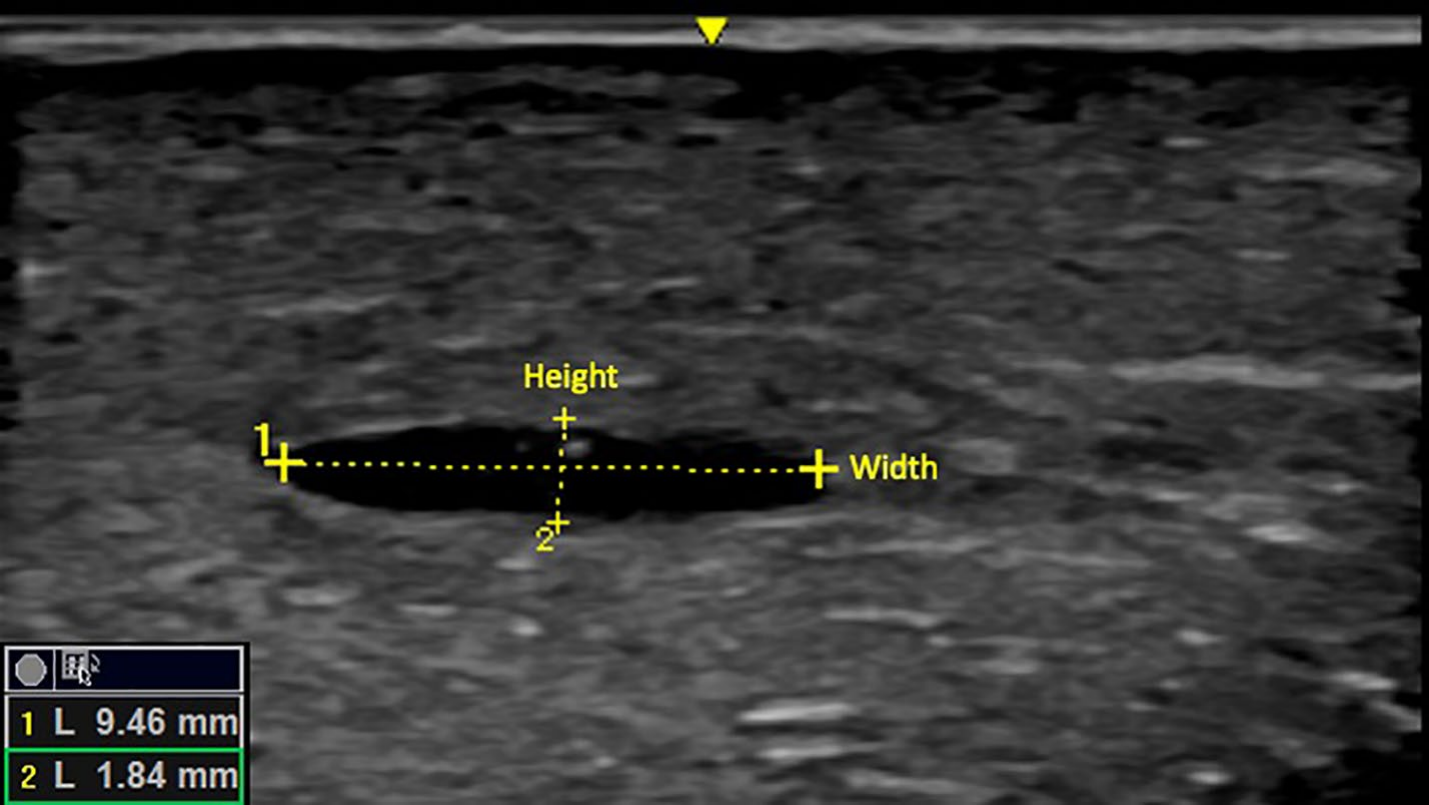
Ultrasound image showing the measurements conducted in this study: Width was defined as the greatest horizontal extent and height was defined as the greatest vertical extent of the soft tissue filler bolus.

Under the model assumption that the injected bolus resembles an ellipsoid, the following equation was used to calculate the volume of the HA bolus:

$$\text{Volume}=\frac{4}{3}\left[\pi \times {\left(\frac{\text{Width}}{2}\right)}^{2}\right]\times \text{Height}$$

To ensure measurement consistency throughout the entire study, all ultrasound‐based measurements were conducted by the same investigator (M.C.). In this study, the ultrasound imaging was conducted using a Logiq e ultrasound system (GE Healthcare, Solingen, Germany) equipped with an L8‐18 MHz hockey stick transducer. To ensure accurate imaging without compressing the tissues, which could lead to measurement artifacts, the transducer was placed in a generous layer of contact gel (Aquasonic Clear Ultrasound Gel, Parker Laboratories Inc., Fairfield, NJ, USA) without direct skin contact.

### 2.3 Statistical analysis

All data were saved in an electronic laboratory notebook (LabArchives, LLC, San Marcos, CA, USA). Differences between groups were calculated using analysis of variance (ANOVA), while differences between follow‐up periods within the investigated groups were determined using paired samples *t*‐test. All calculations were performed using SPSS Statistics 27 (IBM, Armonk, NY, USA), and differences were considered statistically significant at a probability level of ≤0.05 to allow conclusions to be drawn. Results are presented as mean values and their respective 1× standard deviation (mean (SD)).

## 3 RESULTS

### 3.1 General results

Irrespective of the specific soft tissue filler group investigated, the average width and height of the injected HA bolus immediately after the injection measured to be 15.37 mm (1.4) [range: 13.09–17.08] and 4.00 mm (1.1) [range: 2.08–5.80], respectively. 1 h after the application of hyaluronidase, width and height, respectively, were measured to be 11.36 mm (1.3) [range: 10.16–13.75] and 2.53 mm (0.7) [range: 1.43–3.62]. 24 h after the application of hyaluronidase, width and height, respectively, were measured to be 8.74 mm (0.5) [range: 8.10–9.95] and 2.13 mm (0.5) [range: 1.44–3.00].

The calculated volumes amounted to 0.16 cc (0.1) [range: 0.06–0.28], 0.06 cc (0.0) [range: 0.03–0.09], and 0.03 cc (0.0) [range: 0.02–0.04] at baseline, 1 h and 24 h after the injection of hyaluronidase, respectively. No statistically significant differences were found for any of the measurements between the three groups investigated, except for the height measurement at the 1‐h follow‐up (*p* = 0.030) (Table 2).

**TABLE 2:** Summary of the ultrasound‐based measurements of the HA bolus at baseline, 1 hand 24 h after injection of hyaluronidase, stratified for each soft tissue filler group. Statistically significant differences are indicated in bold.

		Group 1			Group 2			Group 3			*p* value
		Mean	SD	Range	Mean	SD	Range	Mean	SD	Range	
Baseline	Width (mm)	16.07	0.69	15.52–16.98	15.15	1.49	13.86–17.08	14.88	1.78	13.09–16.98	0.478
	Height (mm)	4.54	1.15	3.09–5.80	3.96	0.06	3.88–4.01	3.51	1.61	2.08–5.80	0.470
	Volume (cc)	0.20	0.06	0.14–0.28	0.15	0.03	0.13–0.19	0.14	0.10	0.06–0.28	0.489
1 h	Width (mm)	11.32	1.63	10.28–13.75	11.15	0.66	10.61–12.05	11.61	1.64	10.16–13.75	0.896
	Height (mm)	2.75	0.30	2.40–3.12	2.97	0.56	2.28–3.62	1.88	0.59	1.43–2.70	**0.030**
	Volume (cc)	0.06	0.02	0.05–0.09	0.06	0.01	0.06–0.07	0.05	0.00	0.03–0.09	0.470
24 h	Width (mm)	8.55	0.30	8.10–8.77	8.93	0.69	8.42–9.95	8.76	0.49	8.42–9.46	0.595
	Height (mm)	2.51	0.38	2.18–3.00	2.02	0.23	1.78–2.33	1.88	0.52	1.44–2.61	0.111
	Volume (cc)	0.03	0.00	0.02–0.04	0.03	0.00	24.28–29.37	0.02	0.00	0.02–0.03	0.314

### 3.2 Dimensions: Group 1—structuring

The average width and height of the injected product were recorded as 16.07 mm (0.7) [range: 15.52–16.98] and 4.54 mm (1.2) [range: 3.09–5.80] immediately after the soft tissue filler injection, 11.32 mm (1.6) [range: 10.28–13.75] and 2.75 mm (0.3) [range: 2.40–3.12] 1 h after the injection of hyaluronidase and 8.55 mm (0.3) [range: 8.10–8.77] and 2.51 mm (0.4) [range: 2.18–3.00] 24 h after the injection of hyaluronidase, respectively. Statistically significant differences were noted for width between all follow‐up measurements (all *p* < 0.038), while for height only the comparison between the baseline value and the 24‐h follow‐up reached statistical significance (*p* = 0.022).

Immediately after the soft tissue filler injection, the volume of the injected product was calculated to be 0.20 cc (0.1) [range: 0.14–0.28], whereas 1 h after the injection, it amounted to 0.06 cc (0.0) [range: 0.05–0.09] with a 69.7% (3.9) volume reduction (*p* = 0.008). A statistically significant difference was identified between the latter and the 24‐h follow‐up with a 46.5% (11.1) volume reduction to 0.03 cc (0.0) [range: 0.02–0.04] (*p* = 0.035). Between the baseline measurement and the 24‐h follow‐up, a statistically significant volume reduction of 83.7% (3.9) was found (*p* = 0.010).

Within Group 1, the greatest volume reduction after 1 h was seen for Juvedérm Volux with 73.73% while after 24 h it was Belotero Intense with 88.16%. After the initial 1 h postapplication of hyaluronidase, the greatest volume reduction effect was seen for Belotero Intense with 61.21%, indicating a later‐onset effect. (Table 3).

**TABLE 3:** Summary of the ultrasound‐based measurements of the HA bolus at baseline, 1h and 24 h after injection of hyaluronidase, stratified for each soft tissue filler in Group1—Structuring. Statistical significance was defined as *p* values ≤ 0.05.

Group 1–structuring					
		Juvedérm volux	Belotero intense	Restylane lyft	Rennova lift plus
Baseline	Width (mm)	15.55	16.98	16.24	15.52
	Height (mm)	4.27	5.80	3.09	4.99
	Volume (cc)	0.172	0.279	0.136	0.200
1 h	Width (mm)	10.63	13.75	10.28	10.60
	Height (mm)	2.40	2.70	2.76	3.12
	Volume (cc)	0.045	0.085	0.049	0.058
	Volume reduction (to baseline)	73.73%	69.47%	64.21%	70.83%
24 h	Width (mm)	8.10	8.71	8.60	8.77
	Height (mm)	2.24	2.61	2.18	3.00
	Volume (cc)	0.025	0.033	0.027	0.039
	Volume reduction (to baseline)	85.77%	88.16%	80.22%	80.80%
	Volume reduction (to 1 h)	45.81%	61.21%	44.72%	34.18%

### 3.3 Dimensions: Group 2—volumizing

The average width and height of the injected product were 15.15 mm (1.5) [range: 13.86–17.08] and 3.96 mm (0.1) [range: 3.88–4.01] immediately after the soft tissue filler injection, 11.15 mm (0.7) [range: 10.61–12.05] and 2.97 mm (0.6) [range: 2.28–3.62] 1 h after the injection of hyaluronidase and 8.93 mm (0.7) [range: 8.42–9.95] and 2.02 mm (0.2) [range: 1.78–2.33] 24 h after the injection of hyaluronidase, respectively. Statistically significant differences were found for both width and height between all follow‐up measurements (all *p* < 0.035).

Immediately after the soft tissue filler injection, the volume of the injected product was calculated to be 0.15 cc (0.0) [range: 0.13–0.19] and decreased to 0.06 cc (0.0) [range: 0.06–0.07] 1 h after the injection with a 58.9% (9.8) volume reduction (*p* = 0.012). A statistically significant difference was found between the latter and the 24‐h follow‐up with a 55.8% (6.1) volume reduction to 0.03 cc (0.0) [range: 0.02–0.03] (*p* = 0.002). Between the baseline measurement and the 24‐h follow‐up, a statistically significant volume reduction of 82.1% (3.5) was noted (*p* = 0.004).

Within Group 2, the greatest volume reduction after 1 h was seen for Juvedérm Voluma with 69.17%. This filler also showed the greatest volume reduction after 24 h with 86.98%. The smallest volume reduction was seen in Belotero Volume both at 1 h with 47.90% and at 24 h with 78.88%. Interestingly, the greatest volume reduction effect was seen in this filler with 59.46%, indicating a later‐onset effect. (Table 4).

**TABLE 4:** Summary of the ultrasound‐based measurements of the HA bolus at baseline, 1 h and 24 h after injection of hyaluronidase, stratified for each soft tissue filler in Group 2—volumizing. Statistical significance was defined as *p* values ≤ 0.05.

Group 2—volumizing					
		Juvedérm voluma	Belotero volume	Restylane volume	Rennova ultra volume
Baseline	Width (mm)	17.08	14.09	13.86	15.57
	Height (mm)	4.01	3.94	4.01	3.88
	Volume (cc)	0.195	0.130	0.128	0.157
1 h	Width (mm)	10.70	10.61	11.24	12.05
	Height (mm)	3.15	3.62	2.83	2.28
	Volume (cc)	0.060	0.068	0.060	0.055
	Volume reduction (to baseline)	69.17%	47.90%	53.59%	64.80%
24 h	Width (mm)	8.64	8.42	8.71	9.95
	Height (mm)	2.04	2.33	1.92	1.78
	Volume (cc)	0.025	0.028	0.024	0.029
	Volume reduction (to baseline)	86.98%	78.88%	81.09%	81.26%
	Volume reduction (to 1 h)	57.77%	59.46%	59.26%	46.77%

### 3.4 Dimensions: Group 3—lip volumizing

The average width and height of the injected product were measured to be 14.88 mm (1.8) [range: 13.09–16.98] and 3.51 mm (1.61) [range: 2.08–5.80] immediately after the soft tissue filler injection, 11.61 mm (1.6) [range: 10.16–13.75] and 1.88 mm (0.6) [range: 1.43–2.70] 1 h after the injection of hyaluronidase and 8.76 mm (0.5) [range: 8.42–9.46] and 1.88 mm (0.5) [range: 1.44–2.61] 24 h after the injection of hyaluronidase, respectively. Statistically significant differences were found for width between all follow‐up measurements (all *p* < 0.032) without statistically significant differences for any height measurements.

Immediately after the soft tissue filler injection, the volume of the injected product amounted to 0.14 cc (0.1) [range: 0.06–0.28], while it was 0.05 cc (0.0) [range: 0.02–0.09] 1 h after the injection with a 64.0% (16.0) volume reduction, and 0.02 cc (0.0) [range: 0.02–0.03] at the 24‐h follow‐up with a volume reduction of 79.4% (7.1) and 39.5% (20.1) when compared to baseline and the 1‐h follow‐up, respectively. However, no statistically significant differences were obtained for any of the investigated follow‐up measurements.

Within Group 3, substantial variations in terms of volume reduction could be observed. The greatest volume reduction after 1 h was seen for Juvedérm Volift with 76.27% while the smallest volume reduction effect was seen for Rennova lips with 40.38%. The greatest volume reduction after 24 h was seen for Belotero Intense with 88.16%, with the greatest contribution in the time period between 1 h and 24 h, with 61.21%. The smallest overall volume reduction after 24 h was also seen in Rennova lips with 71.36%. (Tables 5 and 6).

**TABLE 5:** Summary of the ultrasound‐based measurements of the HA bolus at baseline, 1 h and 24 h after injection of hyaluronidase, stratified for each soft tissue filler in Group 3—lip volumizing. Statistical significance was defined as *p* values ≤ 0.05.

Group 3—Lip volumizing					
		Juvedérm Volift	Belotero intense	Restylane kysse	Rennova lips
Baseline	Width (mm)	13.77	16.98	15.67	13.09
	Height (mm)	3.28	5.80	2.86	2.08
	Volume (cc)	0.104	0.279	0.117	0.595
1 h	Width (mm)	10.16	13.75	12.00	10.52
	Height (mm)	1.43	2.70	1.47	1.92
	Volume (cc)	0.025	0.085	0.035	0.035
	Volume reduction (to baseline)	76.27%	69.47%	69.86%	40.38%
24 h	Width (mm)	8.43	8.71	9.46	8.42
	Height (mm)	1.61	2.61	1.84	1.44
	Volume (cc)	0.019	0.033	0.027	0.017
	Volume reduction (to baseline)	81.60%	88.16%	76.55%	71.36%
	Volume reduction (to 1 h)	22.49%	61.21%	22.21%	51.95%

**TABLE 6:** Differences between time periods for width, height and volume, stratified for each soft tissue filler group. Statistically significant differences (*p* < 0.05) are highlighted in bold.

		Baseline—1 h	1 h–24 h	Baseline—24 h
		*p* value	*p* value	*p* value
Group 1	Width (mm)	**0.004**	**0.038**	**<0.001**
	Height (mm)	0.051	0.131	**0.022**
	Volume (cc)	**0.008**	**0.035**	**0.010**
Group 2	Width (mm)	**0.016**	**<0.001**	**0.004**
	Height (mm)	**0.035**	**0.011**	**<0.001**
	Volume (cc)	**0.012**	**0.002**	**0.004**
Group 3	Width (mm)	**<0.001**	**0.032**	**0.004**
	Height (mm)	0.075	0.980	0.062
	Volume (cc)	0.076	0.146	0.081

## 4 DISCUSSION

With an increasing number of performed soft tissue filler injections, complications associated with these procedures rise concomitantly. Adverse events can be broadly categorized into nonvascular and vascular complications. Typically, the severity of such complications and their occurrence rates are inversely related: while vascular complications such as visual comprise or tissue necrosis can be dramatic, the likelihood of their occurrence is fortunately low. Beleznay et al. reported a total of 146 cases of vision loss secondary to filler treatments when reviewing the world literature in the time period between 1906 and 2015.^16^, ^17^ On the other hand, nonvascular complications have been reported to be more common and can further be subclassified into early onset and late‐onset complications and include bruising, formation of nodules, and edema among many others.^18^, ^19^, ^20^ In recent years, the scientific community in the aesthetic field has focused more on the prevention, improvement and treatment of such complications arising from soft tissue filler injections. In general, major efforts should be devoted to the prevention of any complications, with key measures involving a thorough knowledge of the injecting clinician in terms of facial anatomy, biomechanics, and basic physiology.^21^, ^22^, ^23^, ^24^ However, if a soft tissue filler‐related complication has occurred, the only option to causally treat this remains the dissolving of the previously applied product via hyaluronidase.^25^ In our study, we investigated the response of different soft tissue fillers to this enzyme, thus broadening our understanding of its capacity to dissolve different products and ultimately resolve complications.

Intriguingly, we herein revealed that, across various ultrasound measurements and time intervals, there was no statistically significant difference among the three soft tissue filler groups—with a single exception which was observed in the measurement of height 1 h postinjection. Notably, Group 3, characterized by the lowest G' values,^6^ exhibited the smallest average height value at this time point. This could be attributed to the reduced firmness of the filler material in this group, potentially allowing for a greater degree of settling or displacement after administration. More importantly, however, the remaining dimensions did not differ significantly, implying that (a) different product characteristics did not influence the shape and behavior of the bolus in the tissue and (b) the fillers did not respond significantly differently after the application of hyaluronidase. In this context, it is important to highlight that we found no significant differences in volume reduction at different follow‐up periods between the investigated groups. This outcome is particularly noteworthy as it suggests that, in the initial stages following filler injections, fillers, in terms of their ability to be dissolved by hyaluronidase, are not influenced by their product characteristics such as G'. Nevertheless, it is should be noted that, in Group 3, which had the lowest average G prime,^6^ the volume measurements did not show statistically significant differences over the compared time intervals (i.e., from baseline to 1 h, from 1 h to 24 h, and from baseline to 24 h). This failure to reach statistical significance might be due to the higher values for standard deviation in this group: this assumption is plausible, as these products exhibit less firmness and, therefore, lose their “original shape” more easily. However, it could also mean that hyaluronidase was less effective in this group, indicating that fillers with a higher G' are more susceptible to the effects of hyaluronidase. The hypothesis of hyaluronidase dissolving soft tissue fillers with high G' values more effectively is further substantiated by the relative volume reduction calculated in this study: albeit not statistically significant, the group with the highest and second highest G' values (i.e., Group 1 and Group 2)^6^ reached the greatest relative volume reductions with 83.7% and 82.1%, respectively, while Group 3 showed a volume reduction of 79.4%.

Of special clinical relevance is the time required to achieve a dissolving effect. Regardless of the soft tissue filler group, the greatest volume reduction, both absolute and relative, was seen in the first hour after the application of hyaluronidase with a 64.1% reduction of the initial volume, whereas, after 24 h, a total volume reduction of 81.7% was noted. Future trials are warranted to investigate the ability to dissolve HA boli after longer times in tissue, such as 1 week or 1 month. Ideally, these studies will be conducted in vivo to establish how the in‐tissue behavior and integration of different products change depending on their properties.

## 5 LIMITATIONS

Firstly, only a selection from the broad market of soft tissue fillers were herein investigated. Our results, therefore, need to be validated in other fillers with similar product characteristics. Secondly, due to the nature of ultrasound imaging, only two‐dimensional images of the HA boli were obtained. Albeit the authors consider their model (which assumes an ellipsoidal shape of the HA boli) to be plausible, this concept must be taken into account when construing the results. Thirdly, it is essential to highlight that chicken breast and living human tissue have different characteristics (for example, varying vasculature, innervation, and temperature). Accordingly, the generalizability, universalizability, and transferability of our results ought to be considered cautiously, with the findings herein presented having to be interpreted in light of the study's inherent limitations. In addition, the interaction of different tissues such as periosteum (e.g., in deep injections) or skin (e.g., in superficial injections) with the injected soft tissue material, was not accounted for. Finally, a longer study period to observe the effects of hyaluronidase on soft tissue filler would have been beneficial to evaluate long‐term outcomes and the presence of any residual amount of product.

## 6 CONCLUSION

Based on the findings of our study, the effectiveness of hyaluronidase in dissolving HA‐based filler is seemingly not influenced by product characteristics such as G‐prime in the initial stages following soft tissue filler injections. The width of the injected HA bolus significantly decreased across all types of fillers studied. Notably, the volume reduction was particularly significant in the two filler groups with the highest and second‐highest G‐prime values, while the filler group with the lowest G' value showed no statistically significant differences over the follow‐up period, suggesting that hyaluronidase is more effective in fillers with higher G‐prime values.

## AUTHOR CONTRIBUTIONS

Bruna S. F. Bravo is a medical consultant and speaker for Rennova®, Allergan Aesthetics, Merz Aesthetics®, and L'Oréal. Thamires S. C. de Almeida, Camila Silveira, Maria Carolina Zafra, and Mariana Calomeni Elias are medical speakers for Rennova®.

## FUNDING INFORMATION

The authors received financial support from Rennova® for the research and publication of this article. The products utilized in this study were donated by the injectors for the purposes of this study.

## ETHICS STATEMENT

This study was exempt from ethics approval as no living tissue was investigated.

## Data Availability

The data that support the findings of this study are available from the corresponding author upon reasonable request.
